# Effect of Acetazolamide on Postural Control in Patients with COPD Travelling to 3100 m Randomized Trial

**DOI:** 10.3390/jcm12041246

**Published:** 2023-02-04

**Authors:** Aline Buergin, Michael Furian, Laura Mayer, Mona Lichtblau, Philipp M. Scheiwiller, Ulan Sheraliev, Talant M. Sooronbaev, Silvia Ulrich, Konrad E. Bloch

**Affiliations:** 1 Department of Respiratory Medicine, University Hospital Zurich, Rämistrasse 100, 8091 Zurich, Switzerland; 2 Swiss-Kyrgyz High Altitude Medicine and Research Initiative, 8091 Zurich, Switzerland; 3 Swiss-Kyrgyz High Altitude Medicine and Research Initiative, Bishkek 720040, Kyrgyzstan; 4 Department of Respiratory Medicine, National Center for Cardiology and Internal Medicine, Bishkek 720040, Kyrgyzstan

**Keywords:** postural control, high-altitude, COPD, acetazolamide, hypoxia, hypoxemia

## Abstract

Patients with chronic obstructive pulmonary disease (COPD) may be susceptible to impairments in postural control (PC) when exposed to hypoxia at high altitude. This randomized, placebo-controlled, double-blind, parallel-design trial evaluated the effect of preventive acetazolamide treatment on PC in lowlanders with COPD traveling to 3100 m. 127 lowlanders (85 men, 42 women) with moderate to severe COPD, aged 57 ± 8 y, living below 800 m, were randomized to treatment with acetazolamide 375 mg/d starting 24 h before ascent from 760 m to 3100 m and during a 2-day sojourn in a clinic at 3100 m. PC was evaluated at both altitudes with a balance platform on which patients were standing during five tests of 30 s each. The primary outcome was the center of pressure path length (COPL). In the placebo group, COPL significantly increased from (mean ± SD) 28.8 ± 9.7 cm at 760 m to 30.0 ± 10.0 cm at 3100 m (*p* = 0.002). In the acetazolamide group, COPL at 760 m and 3100 m were similar with 27.6 ± 9.6 cm and 28.4 ± 9.7 cm (*p* = 0.069). The mean between-groups difference (acetazolamide-placebo) in altitude-induced change of COPL was −0.54 cm (95%CI −1.66 to 0.58, *p* = 0.289). Multivariable regression analysis confirmed an increase in COPL of 0.98 cm (0.39 to 1.58, *p* = 0.001) with ascent from 760 to 3100 m, but no significant effect of acetazolamide (0.66 cm, 95%CI −0.25 to 1.57, *p* = 0.156) when adjusting for several confounders. In lowlanders with moderate to severe COPD, an ascent to high altitude was associated with impaired postural control and this was not prevented by acetazolamide.

## 1. Introduction

Intact postural control (PC) is essential for safe performance of many everyday activities including professional work and leisure activities. PC is a complex function of the body that requires visual, somatosensory, and vestibular inputs; the central nervous system for their integration; and the control of skeletal muscles [1]. Hypoxia may affect these processes within a few minutes [2]. Therefore, people who ascend to higher altitudes may suffer not only from acute altitude-related illness, but also from other altitude-related adverse health effects (ARAHE), including PC impairment.

Only few studies have evaluated PC at high altitude. In healthy individuals, worsening of PC has been shown in hypobaric chambers [3,4] as well as in field studies at high altitudes of 3619 to 5140 m [5,6] and at moderately high altitudes of 1650 to 2590 m, corresponding to that of many tourist destinations [7]. PC may be affected directly by hypoxia and indirectly by symptoms of acute mountain sickness (AMS) such as headache, weakness, dizziness, and ataxia, which may impair concentration.

Chronic obstructive pulmonary disease (COPD) is a highly prevalent disease characterized by chronic inflammation of the airways and parenchymal destruction of the lung, resulting in airflow obstruction as well as impaired pulmonary gas exchange [8]. Patients with COPD have been shown to have an impaired PC near sea level associated with physical inactivity, muscle weakness, and advanced age [9]. This impairment, however, seems not to be improved with supplemental oxygen [10]. During high altitude travel, patients with COPD may be particularly susceptible to impairments in PC as their lung disease may result in more pronounced hypoxemia at a given altitude compared to healthy individuals. Consistently, in lowlanders with COPD, we observed significantly increased postural instability measured by a balance board at 3100 m [11]. For prevention of dangerous events, such as falls, especially at high altitude with little access to healthcare facilities, it would be desirable to have an effective means to prevent the risks of PC instability. Therefore, the purpose of the current study was to test the hypothesis that acetazolamide, a carbonic anhydrase inhibitor recommended for prevention of AMS in healthy mountain travelers [12], mitigates altitude-related impairments in PC in patients with COPD during a stay at 3100 m.

## 2. Materials and Methods

### 2.1. Study Design and Setting

This study was performed as part of a randomized, placebo controlled, double blind parallel-design trial, evaluating the efficacy of acetazolamide in reducing the incidence of ARAHE in lowlanders with COPD during a stay for 2 days at the high-altitude clinic Too Ashu, 3100 m, Kyrgyz Republic. ARAHE was defined as either severe AMS, severe hypoxemia, or any other symptoms requiring intervention or treatment as well as study withdrawal upon request by the patient or the supervising independent physician. Participant characteristics and data on ARAHE have been reported elsewhere [13], data on PC, the focus of the current nested trial, have not been published.

### 2.2. Subjects

We included Kyrgyz men and women, aged 20–75 years, living below 800 m and diagnosed with COPD with an FEV1/FVC <0.7, and an FEV1 between 40% and 80% predicted, consistent with grades 2–3 according to the Global Initiative for Chronic Obstructive Lung Disease (GOLD) guidelines [8]. Exclusion criteria were pulse oximetry (SpO_2_) <92% or hypercapnia PaCO_2_ >6.0 kPa while breathing ambient air at rest during a baseline evaluation at 760 m, COPD exacerbation, current infectious disease, uncontrolled cardiovascular disease (arterial hypertension, coronary artery disease, previous stroke) or diabetes, a history of obstructive sleep apnea, long-term oxygen therapy, and other conditions that might have interfered with the protocol such as alcoholism or current heavy smoking (>20 cigarettes per day).

### 2.3. Interventions

Baseline measurements took place at the National Center of Cardiology and Internal Medicine, in Bishkek (760 m). Subsequently, patients ascended in a minibus within 5–6 h to the Too Ashu high altitude clinic at 3100 m where they stayed for 2 days and nights. Starting 24 h before ascent and during the stay at 3100 m, participants were administered the study medication under supervision of an investigator. It consisted of acetazolamide capsules (125 mg) or identically looking placebo capsules, one in the morning and two before sleep. The total daily dose of acetazolamide was 375 mg.

### 2.4. Assessments

Evaluation of postural control: In all participants, PC was assessed at both altitudes on the second day in the morning, i.e., under the influence of the study drug, using a Wii Balance Board (WBB, Redmond, WA, USA), previously validated and described in detail [14,15]. In a subgroup of 65 patients, PC protocol was additionally assessed before the first dose of the study drug to evaluate the effect of acetazolamide at low altitude. PC assessments consisted in a series of five identically performed balance tests of 30 s duration each whilst the center of pressure path length (COPL) of the body was recorded. To this end, patients were standing on the board with eyes open and feet in a 30° angle. They were instructed to stand as still as possible during the tests and to keep their arms beside the body while fixing their eyes on a black dot, 2 cm in diameter, placed at eye level on a wall at a distance of 1.5 m. For the weekly calibration of the WBB, as well as for computing the PC variables, a customized software was used (Labview 8.5 National Instruments, Austin, TX, USA) [14]. For a greater repeatability, we always used the same device and performed the tests at the same time of day, as suggested previously [16,17]. The COPL was the main outcome, sway velocities and amplitudes in both the antero-posterior and medio-lateral plane were additional PC outcomes.

Additional assessments: A medical history was obtained, and clinical examinations were performed. AMS was assessed by the Lake Louise Score (LLS) [18] as well as by the environmental symptoms cerebral score (AMSc) [19]. A Lake Louise score of ≥3 in the presence of headache or an AMSc score ≥0.7 were considered as clinically relevant AMS [20]. Pulse oximetry (Konica-Minolta PULSOX-300i, Anandic Medical Systems AG, Feuerthalen, Switzerland), arterial blood gases (RapidPoint, Siemens, Munich, Germany), spirometry (BlueCherry, Geratherm Respiratory, Bad Kissingen, Germany), exercise tests (6-min walk and maximal cycling exercise), as well as sleep studies (Alice PDx, Philips Respironics, Murrysville, PA, USA) were performed with their data being reported elsewhere [13].

ARAHE were defined as one or several of the following: presence of severe AMS symptoms (AMSc ≥ 0.7 or LLS ≥ 3), severe hypoxemia at rest (SpO_2_ < 80% for >30 min, SpO_2_ < 70% for >15 min), or any condition requiring study withdrawal according to the decision of an independent physician or the wish of the patient. For safety reasons, patients with ARAHE were treated with oxygen or other means and transported to lower altitude as appropriate. Side effects including polyuria, tingling sensation, and change in taste were assessed throughout the stay at 3100 m.

### 2.5. Outcomes and Sample Size Estimation

The primary outcome was the COPL. As the minimal clinically important difference in COPL in COPD patients has not been established, the study was powered with 80% to detect a moderate effect size of 0.5, alpha 0.05, indicating a required number of 128 participants.

### 2.6. Randomization and Blinding

The 1:1 randomization to either one of the drugs was made with a computer-generated schedule minimizing for sex, age (≤50 or >50 years), and FEV1 predicted (<60% or ≥60%) (MinimPy 0.3, Distributed under the GNU GPL v3) [21]. An independent pharmacist prepared active and identically looking placebo capsules labelled with a secret 4-digit number. The key assigning numbers to either placebo or acetazolamide was not known to participants and researchers until data analysis was completed.

### 2.7. Data Analysis

The primary data analysis was performed in the intention to treat population of all participants who underwent general baseline measurements at lowland and at least one balance board measurement. Missing values and values from subjects receiving O_2_ at altitude in the night before the balance test were replaced by multiple imputation. Additionally, a per protocol analysis was conducted among participants with complete data. The effects of altitude and treatment were evaluated by computing mean differences and 95% confidence intervals (95%CI) over all 10 tests with a multivariate regression analysis. Additionally, the effect of several independent parameters (presence of AMS, age, gender, weight, or height) on COPL was examined. As most of the data was non-parametric, COPL as well as the sway velocities were transformed to 1/square root of the respective variable. Sway amplitudes were log transformed. Effect sizes were quantified by Cohen’s d with d = difference between two means (3100 m vs. 760 m or change with altitude placebo vs. change with altitude acetazolamide) divided by the pooled standard deviation (SD). Values for d < 0.2 are considered small, 0.5 to 0.8 medium and >0.8 strong effects [22]. A probability of <0.05 was considered statistically significant.

## 3. Results

### Study Population

**Figure 1:**
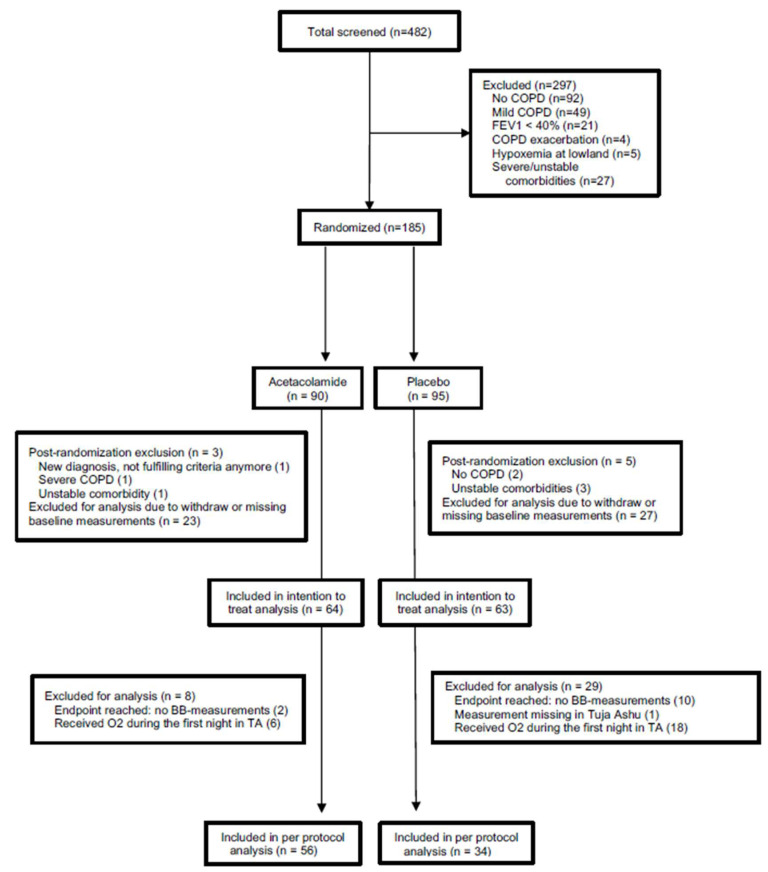
Flow chart: BB = Balance Board; TA = Too Ashu high altitude clinic at 3100 m.

Of 185 randomized patients, some did not fulfill the inclusion criteria during later review or had missing baseline PC measurements and were, therefore, excluded post-randomization, leaving 127 patients for the intention to treat analysis (Figure 1). There was no statistical significance regarding demographic characteristics between the two groups. The mean age of the 127 patients was 57.4 ± 8.3 years (ranging from 34 to 74 years). AMS was present in 46 of the 127 patients (36%), 25 (40%) under placebo and 21 (33%) under acetazolamide treatment (Table 1). Acetazolamide was generally well tolerated although participants in the acetazolamide group more commonly reported tingling sensations of at least mild intensity compared to the placebo group (Table 1).

The altitude induced increase in COPL in the placebo group from (mean ± SD) 28.78 ± 9.68 cm to 30.04 ± 9.95 cm was statistically significant (*p* = 0.002), whereas this was not the case for the acetazolamide group, with an increase from 27.64 ± 9.61 cm to 28.38 ± 9.65 cm (*p* = 0.069). The antero-posterior sway amplitude was significantly increased at 3100 vs. 760 m only in the acetazolamide group (*p* < 0.001, Table 2) while the antero-posterior sway velocity was significantly enhanced at altitude in both groups. The medio-lateral sway amplitude was improved at altitude in the placebo group (Table 2). The mean effect size (Cohen’s d) of the altitude-induced changes in COPL were small in both groups, i.e., d = 0.14 in the placebo and d = 0.08 in the acetazolamide group.

**Figure 2:**
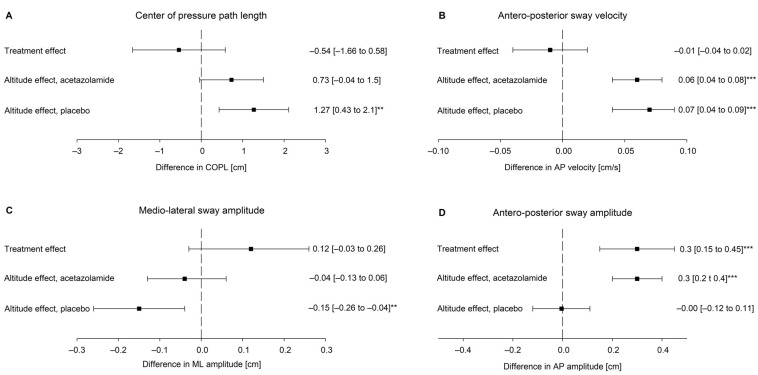
Postural control at 760 m and 3100 m with placebo and acetazolamide treatment. Mean within-group changes (95% confidence intervals) with ascent from 760 m to 3100 m and between-group differences in changes (95% confidence intervals) are shown for (**A**) Centure of pressure path lengt; (**B**) Antero-posterior sway velocity; (**C**) Medio-lateral sway amplitude; (**D**) Antero-posterior sway amplitude. ** *p* < 0.01; *** *p* < 0.001.

Figure 2 shows that the mean difference between acetazolamide and placebo in altitude-induced changes was non-significant regarding COPL with −0.54 cm (95%CI −1.66 to 0.58, *p* = 0.289), whereas worsening of the altitude-induced change regarding antero-posterior sway amplitude was greater with acetazolamide by 0.3 cm (95%CI 0.15 to 0.45, *p* < 0.001) compared to placebo, with mean effect size of d = 0.44. Acetazolamide had no significant effect on the sway velocities (Supplementary Table S1, per protocol analysis).

When adjusting COPL for several potential confounders, the significant effect of altitude was confirmed and a significant association between body height as well as AMSc and increased COPL was found (Table 3), while the drug effect remained non-significant.

When comparing data from lowland with and without drug, controlled for potential confounders, a significant improvement could be found with placebo compared to no drug for both antero-posterior (*p* = 0.002) and medio-lateral sway amplitude (*p* = 0.019). A significant difference in drug-induced changes between both drugs, however, was only seen in the antero-posterior plane, where acetazolamide diminished the improvement seen under placebo compared to no drug (*p* = 0.015) (Table 4).

## 4. Discussion

In this randomized, placebo controlled, double-blind study, we investigated the effects of high altitude (3100 m) and preventive acetazolamide treatment on postural control (PC) in lowlanders with moderate to severe COPD (GOLD grades 2–3). We found PC to be impaired at the higher altitude with an increased COPL and antero-posterior sway velocity. Acetazolamide did not prevent the altitude-induced impairment of PC.

The current study is the first investigating the effect of acetazolamide on PC in lowlanders with COPD travelling to high altitude. Whereas previous studies regarding PC under hypoxic conditions addressed mainly young, healthy mountaineers, Muralt and coworkers [11] were the first to show an altitude-related increase in COPL and antero-posterior velocity in mild to moderate COPD patients. The predominance of an altitude-induced impairment in control of body movements in the antero-posterior over the medio-lateral plane was also observed in the current study, which was consistent with previous studies in healthy individuals in various settings and with different devices [3,6,7,23].

The control of motion in the antero-posterior plane seems not only to be vulnerable to hypoxia, but also to respiratory movements as seen in healthy subjects with a voluntarily increased tidal volume [24] and in COPD patients with an elevated respiratory rate [25]. These authors related the PC disturbance to a greater abdominal muscle activity during forced breathing, concomitant with a stiffness of the trunk. Stimulation of ventilation by acetazolamide may be one explanation of the larger antero-posterior sway amplitude observed with acetazolamide compared to placebo in the current study. Even though we did not measure arterial blood gases or ventilation rates during balance tests, enhanced ventilation in the acetazolamide group is suggested by a lower PaCO_2_ and higher SpO2 at 3100 m compared to the placebo group measured before PC testing [13].

Consistent with our results in a subgroup at 760 m, Collier and coworkers [26] observed an increased postural sway in healthy subjects under acetazolamide compared to placebo. Additionally, they proved the drug to cause modest central nervous system side effects, such as dizziness and impaired concentration, in accordance with other studies indicating impaired cognitive function as well as delayed reaction time already under small doses of 125 mg acetazolamide per day [27]. PC variables, in particular the antero-posterior sway [28], were found to correlate with cognition. Taking all this into account, our observed deterioration in PC under acetazolamide vs. placebo at low altitude (760 m) might be explained by the drug’s negative effects on neuropsychological functions [29]. In conclusion, in the current study acetazolamide seemed to attenuate an improvement in the antero-posterior sway amplitude observed under placebo, but if it actually impairs PC compared to not taking any drug needs further investigation.

Studies testing possible measures for prevention or treatment of an altitude-impaired PC are rare. Our recent study on the efficacy of dexamethasone in preventing postural instability in COPD patients at 3100 m failed to show a beneficial effect but also no harm [11]. In addition, supplemental oxygen given to healthy subjects at 4559 m revealed no positive effect on PC, only on AMS [23]. The latter fact caused the authors to attribute postural ataxia at altitude to other hypoxia-dependent mechanisms than AMS. It was shown that postural instability diminishes after some time at altitude [30], but the time required for such an acclimatization is unclear, since the impairment persisted during 3 days in the study of Baumgartner et al. [5]. 

Although the AMS questionnaire evaluates dizziness and loss of coordination, as well as heel-to-toe walking, as a measure of ataxia [19,31], data on the correlation of postural instability at altitude and AMS are scant, mostly showing no relation [4,5,6]. Our data showed a significant association of the AMSc score at time of the balance test with the COPL. However, this was not true for any other PC variable or when taking the LLS instead of AMSc, and hence, this might be a spurious finding.

Besides the AMSc, we found body height to adversely affect COPL, but surprisingly not age, as the data from Muralt and coworkers suggested [11]. This might be because we additionally included baseline values of PC in our model, which already showed to be significantly affected by age (*p* = 0.009). However, literature about PC and age is controversial [32].

One limitation of our study is that we used multiple imputation for missing data in the intention to treat analysis even though data loss might not have been missing completely at random as patients under placebo experienced more ARAHE. However, findings were confirmed in the per protocol population. Further, despite recovery periods between exercise tests and postural control assessments, we cannot rule out carry-over effects of physical activity [33]. However, to reduce this bias, subjects strictly maintained a break of 30 min after each exercise test. We cannot directly correlate our results of altitude-induced PC-impairment with a clinical outcome, such as falls, but different PC variables, assessed either with the WBB [34,35] or other force-platforms [36], were shown to correlate with future falls. For example, Johansson and coworkers [35] showed a nearly twofold (OR = 1.9) risk of future falls in 70-year-old individuals with longer COPL (i.e., at the highest quintile) compared to those in the first quintile. For comparison, we found an altitude-related increase of COPL of >20% in 20 (16%) COPD patients.

## 5. Conclusions

This study demonstrates that lowlanders with moderate to severe COPD (FEV1 40–80% predicted) experience impaired PC with increased COPL and antero-posterior sway velocity. Acetazolamide has no beneficial effect on these outcomes. Further studies on measures for preventing postural instability at altitude are warranted.

## Figures and Tables

**Table 1:** Patient characteristics.

		Placebo Group	Acetazolamide Group	All Participants
N		63	64	127
Sex, male/female		44/19	41/23	85/42
Age, y		58.40 ± 9.34	56.50 ± 7.13	57.4 ± 8.32
Weight, kg		72.63 ±14.55	74.42 ± 12.39	73.53 ± 13.48
Height, m		1.64 ± 0.08	1.65 ± 0.09	1.64 ± 0.08
BMI, kg/m²		27.06 ± 4.95	27.29 ± 4.01	27.18 ± 4.49
Cigarettes, pack years		20.1 ± 26.5	13.7 ± 16.1	16.9 ± 22.1
**Assessments at baseline (760 m)**				
FEV1, % predicted		60 ± 13	60 ± 11	60 ± 12
SpO_2_, %		94 ± 2	94 ± 2	94 ± 2
**Assessments at 3100 m**				
SpO_2_, %		88 ± 3	89 ± 3 *	89 ± 3
AMS, *n*		25 (40%)	21 (33%)	46 (36%)
Side effects (*n*, %)	Tingling sensation	2 (3%)	11 (17%) *	
	Polyuria	10 (16%)	17 (27%)	
	Change in taste	3 (5%)	2 (3%)	
	Other	1 (2%)	1 (2%)	

Values are presented as mean ± SD; * *p* < 0.05 acetazolamide versus placebo; BMI = Body mass index; FEV1 = Forced expiratory volume in 1 s; SpO_2_ = Arterial oxygen saturation, AMS = Acute mountain sickness.

**Table 2:** Postural control at 760 m and 3100 m with placebo and acetazolamide treatment.

	Placebo		Acetazolamide	
	760 m	3100 m	760 m	3100 m
Center of pressure path length, cm	28.78 ± 9.68	30.40 ± 9.95 **	27.64 ± 9.61	28.38 ± 9.65
Antero-posterior amplitude, cm	2.27 ± 0.81	2.27 ± 0.90	2.19 ± 0.81	2.49 ± 0.82 **
Antero-posterior velocity, cm/s	0.71 ± 0.26	0.78 ± 0.27 **	0.69 ± 0.26	0.74 ± 0.26 **
Medio-lateral amplitude, cm	1.68 ± 0.69	1.53 ± 0.77 **	1.52 ± 0.69	1.48 ± 0.69

Values are presented as mean ± SD; ** *p* < 0.01 vs. baseline at 760 m.

**Table 3:** Effect of high-altitude exposure on the center of pressure path length evaluated by multivariable regression analysis.

Dependent Variable: Center of Pressure Path Length, cm			
	Coefficient	95%CI	*p*-Value
Altitude (1 = 760 m; 2 = 3100 m)	0.98	0.39 to 1.58	0.001
Drug (1 = Plc, 2 = AZA)	0.66	−0.25 to 1.57	0.156
Age, years	−0.002	−0.06 to 0.05	0.937
Sex (1 = men; 2 = women)	0.59	−0.84 to 2.01	0.422
Height, cm	0.1	0.01 to 0.19	0.030
Weight, kg	−0.04	−0.08 to 0.01	0.084
AMSc (at morning of PC test)	2.74	0.42 to 5.06	0.021
COPL baseline (at 760 m)	0.87	0.79 to 0.94	<0.001
Intercept	−11.58	−27.47 to 4.31	0.153

Plc = Placebo; AZA = Acetazolamide; COPL = center of pressure path length; AMSc = acute mountain sickness environmental symptom score assessed at the morning of postural control test.

**Table 4:** Sub analysis—postural control with drug compared to without drug at lowland.

	Placebo		Acetazolamide		Difference in Changes between Groups
	Without Drug	With Drug	Without Drug	With Drug	
COPL, cm	30.16 ± 9.00	29.49 ± 9.01	26.50 ± 9.14	26.40 ± 9.13	0.48 [−1.17 to 2.13]
AP amplitude, cm	2.36 ± 0.76	2.08 ± 0.77 **	2.25 ± 0.77	2.22 ± 0.77	0.27 [0.07 to 0.48] ^##^
AP velocity, cm/s	0.75 ± 0.24	0.74 ± 0.24	0.65 ± 0.25	0.66 ± 0.25	0.02 [−0.02 to 0.07]
ML amplitude, cm	0.52 ± 0.17	0.49 ± 0.17 *	0.46 ± 0.17	0.45 ± 0.17	0.09 [−0.08 to 0.26]

Values are presented as mean ± SD and mean differences with 95% confidence intervals; * *p* <0.05/** *p* < 0.01 vs. baseline without drug, ^##^ *p* < 0.01 change with acetazolamide vs. change with placebo. Differences and their *p* values are adjusted for age, sex, height, weight, baseline values of PC measurements, FEV1 at 760 m, smoking and AMSc-score. COPL = center of pressure path length; AP = antero-posterior; ML = medio-lateral.

## Data Availability

Anonymized data underlying this study can be requested by qualified researchers providing an approved proposal.

## Supplementary Materials

The following supporting information can be downloaded at: https://www.mdpi.com/article/10.3390/jcm12041246/s1, Table S1: Postural control at 760 m and 3100 m with placebo and acetazolamide treatment; per protocol analysis (*n* = 90).
