# Supplementary material for: Effect of Acetazolamide on Postural Control in Patients with COPD Travelling to 3100 m Randomized Trial
Source: J Clin Med. 2023 Feb 4;12(4):1246. doi: 10.3390/jcm12041246 (PMC9960941; doi:10.3390/jcm12041246)
Supplement: Supplementary file 1 [file jcm-12-01246-s001.zip › jcm-2043652-supplementary.pdf]

Supplementary Table S1. Per-protocol analysis

	Placebo		Acetazolamide		Difference in altitude-induced changes between groups
	760 m	3100 m	760 m	3100 m	
COPL, cm	28.06 ± 11.17	29.34 ± 11.17 *	27.35 ± 8.7	28.34 ± 8.7 *	0.48 [-1.17 to 2.13]
AP amplitude, cm	2.26 ± 0.93	2.17 ± 0.93	2.12 ± 0.73	2.47 ± 0.73 **	0.27 [0.07 to 0.48] ##
AP velocity, cm/s	0.69 ± 0.31	0.75 ± 0.31 **	0.68 ± 0.24	0.74 ± 0.24 **	0.02 [-0.02 to 0.07]
ML amplitude, cm	1.62 ± 0.72	1.47 ± 0.72 *	1.48 ± 0.56	1.48 ± 0.56	0.09 [-0.08 to 0.26]

Values are presented as mean ± SD and mean differences with 95% confidence intervals, n=90; *p <0.05 / **p <0.01 vs. baseline without drug, ##p <0.01 change with acetazolamide vs. change with placebo.
Differences and their p values are adjusted for age, sex, height, weight, baseline values of PC measurements, FEV1 at 760m, smoking and AMSc-score.
COPL = center of pressure path length; AP = antero-posterior; ML = medio-lateral
